# An Approach for In Situ Rapid Detection of Deep-Sea Aromatic Amino Acids Using Laser-Induced Fluorescence

**DOI:** 10.3390/s20051330

**Published:** 2020-02-29

**Authors:** Ranran Du, Dingtian Yang, Guangjia Jiang, Youren Song, Xiaoqing Yin

**Affiliations:** 1 Guangdong Key Lab of Ocean Remote Sensing, State Key Laboratory of Tropical Oceanography, South China Sea Institute of Oceanology, Chinese Academy of Sciences, Guangzhou 510301, China; duranran@scsio.ac.cn (R.D.);; 2 University of Chinese Academy of Sciences, Beijing 100049, China; 3 Southern Marine Science and Engineering Guangdong Laboratory, Guangzhou 511458, China; 4 South China Sea Environment Monitoring Center, State Oceanic Administration, Guangzhou 510300, China

**Keywords:** soluble aromatic amino acid, laser-induced fluorescence, in situ detection

## Abstract

Amino acids are the material basis of almost all life activities. An improved understanding of the source, state, and cycle of amino acids is essential for determining the energy flow and material circulation of marine ecosystems. In the present study, an in situ rapid detection method of ultraviolet (UV; 266 nm) laser-induced fluorescence (LIF) technology was used to detect three natural, aromatic amino acids in the seawater. The laser-induced fluorescence peaks of aromatic amino acids tryptophan, tyrosine, and phenylalanine were located at 350 nm, 300 nm, and 280 nm, respectively. High, linear correlations between the concentrations of the aromatic amino acids and the fluorescence peak heights were observed, and the lowest detectable concentrations of tryptophan, tyrosine, and phenylalanine were 4.70 × 10^−9^ mol/L, 2.76 × 10^−8^ mol/L, and 6.05 × 10^−7^ mol/L, respectively, which allowed us to quantify their concentrations by using laser-induced fluorescence. This paper not only provides a practical method for the detection of aromatic amino acids in seawater, but a new means to further understand the biogeochemical processes of carbon cycles in the deep sea.

## 1. Introduction

**Figure A1:**
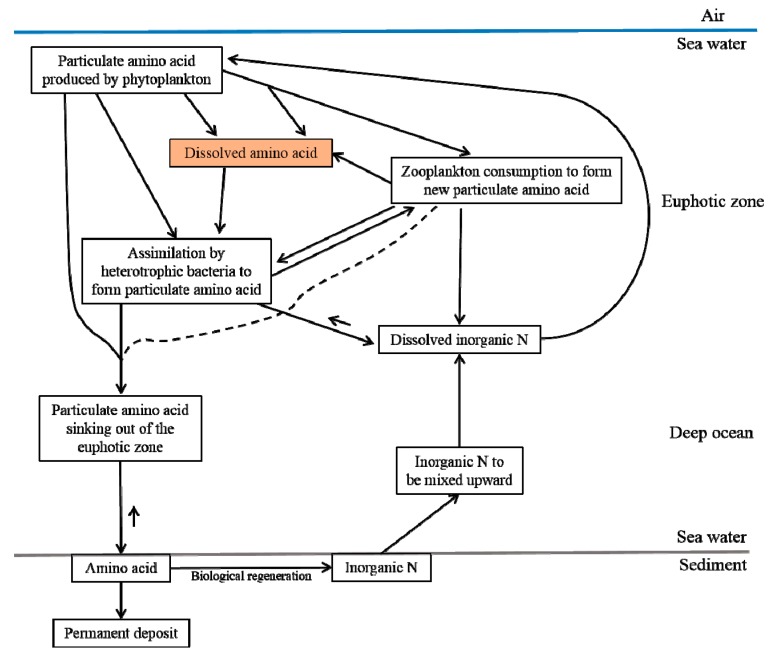
Simplified schematic of the marine organic nitrogen cycle [48].

Amino acids, a class of organic compounds containing amino and carboxyl groups, make up the basic structure of protein macromolecules, and almost all life activities are related to them. In the natural world, there are more than 300 kinds of amino acids, which can be divided into protein and nonprotein amino acids. It is reported that about 20 amino acids are very important components of biological proteins [1]. In the ocean, amino acids are also the main components of the carbon pool and are important organic carbon inputs for the underwater sedimentary environment [2,3]. Amino acids also play an important role in the global nitrogen cycle and the biogeochemical cycle of organic materials (see Figure A1 in Appendix A). Total hydrolyzed amino acids (THAAs) account for about 10%–20% of dissolved organic nitrogen (DON) in the ocean, which dominates the nitrogen sources for microbes in the photic zone [4,5,6]. Some researchers have observed that amino acids are good indicators of the degradation behavior of particulate organic matter (POM) [7,8], dissolved organic matter (DOM) [9], and sedimentary organic matter (SOM) [10,11]. Zhang et al. [12,13] used tryptophan and tyrosine as parallel factors to evaluate the environmental dynamics (i.e., source and fate) of DOM in the surface water. Dauwe et al. [14] confirmed that amino acids such as phenylalanine, tyrosine, and isoleucine become depleted with increased degradation states. Furthermore, dissolved free amino acids (DFAAs) are excellent indicators of the biological processes of DOM in seawater [15,16]. Other findings indicate that the content and composition of aromatic amino acids are responsible for the nutrient status and ecological types of lakes, thus acting as biomarkers [17]. Moreover, the concentration of amino acids in water is related to algal blooms [18,19]. For example, Horiuchi et al. expressed that the amino acid ratio of D-enantiomers to L-enantiomers (D/L) can be used as a biomarker to verify the microbial activity in hydrothermal environments [20]. Additionally, the aromatic amino acids tryptophan, tyrosine, and phenylalanine, constitute 1%–5% of the dry weight of some typical bacteria [21]. Tryptophan is an amino acid in all living cells and has a well-defined fluorescence spectrum [22], which has been used to detect certain diseases [23,24]. In oceanic ecosystems, the accurate detection of amino acids will highly improve their significance in understanding the biogeochemical cycles of organic matter and marine nitrogen pools [25].

The importance of amino acids in the ocean environment and their usages as indicators of some marine activities have attracted more attention in recent years. However, as a result of the lack of suitable detection methods, limited research on amino acids in seawater has been performed. As mentioned by Mopper et al. [26], in addition to the problems and deficiencies in the amino acid detection methods, the problems related to sample handling, such as the effects of sample filtration and short-term storage, have not been completely solved. In 1966, Sigel developed a ligand ion exchange method that can concentrate low concentrations of amino acids in seawater to a certain degree, and can be used for analysis with traditional methods [27]. In recent years, more techniques for detecting amino acids in the ocean have been developed, such as chemical, electrochemical, spectrophotometric, and chromatographical methods (see Appendix B). High performance liquid chromatography (HPLC) is the most common method for analyzing amino acid components, and it is widely used because of its high detection accuracy in identifying compounds [28,29]. However, the HPLC detection method is time-consuming and labor-intensive, with some measurement errors arising from derivatized impurities in the sample [30,31,32,33].

Compared with routine HPLC for amino acid analysis and its coupling with other instruments, laser-induced fluorescence (LIF) has advantages for in situ detection and has the ability to run a long-term, uninterrupted operation that can detect and collect sample information 100 times per second in order to obtain varying temporospatial data [34] for amino acids. Additionally, a series of processes caused by ex situ observations, which can change a sample’s amino acid concentration, can be avoided. We believe that the in situ measurement of water bodies will inevitably become one of the key development directions for water environment detection in the future. Furthermore, LIF equipment can be used as a supplement to traditional amino acid detection technologies in order to make up for the lack of in situ detection. In this paper, a suitable device for UV laser-induced fluorescence was developed to detect aromatic amino acids in situ in the ocean, which, in the future, could be used to study aromatic amino acid concentration and distribution, and trace aromatic amino acid cycles in deep-sea marine ecosystems.

## 2. Material and Methods

### 2.1. Instrument Design

**Figure 1:**
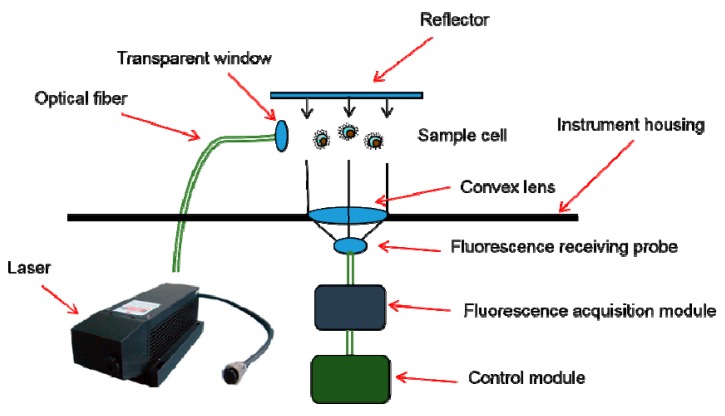
Schematic of a laser-induced fluorescence (LIF) system.

**Figure 2:**
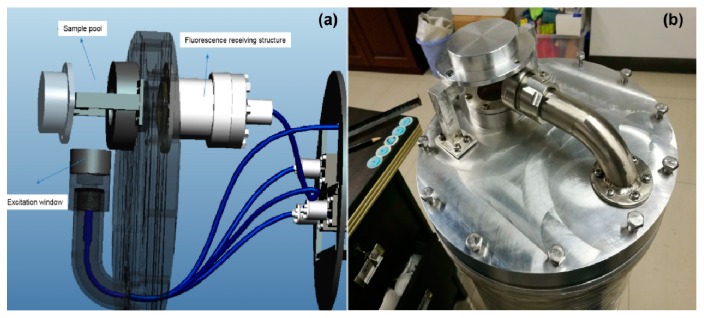
Sample pool design, where (**a**) is a simulated perspective view and (**b**) is a prototype.

**Figure 3:**
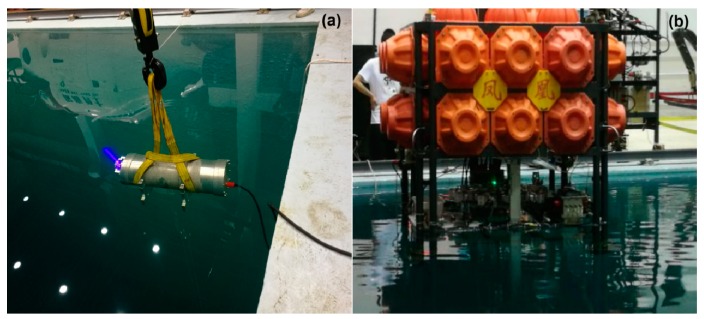
Real-time in situ detection with an LIF instrument. (**a**) The LIF instrument works in the pool and (**b**) The LIF instrument is carried on the Phoenix underwater work platform.

The principle of the instrument for the in situ deep-sea detection of aromatic amino acids is shown in Figure 1. Having considered the particularity of the underwater environment [35], we specially designed the LIF instrument (to be waterproof, pressure-resistant, corrosion-resistant, etc.). The UV laser emitted light through optical windows to directly excite seawater, and the laser-induced fluorescence of the seawater was gathered and transferred in the form of electrical signals through a photoelectric conversion module. The fluorescence peaks of the electrical signals were used to classify the aromatic amino acids and to analyze their concentrations. In order to simplify the instrument structure and save more energy in the deep sea, an open sample pool was designed to avoid using a pump (Figure 2). The working distance of the instrument was 10 cm. In order to detect weak fluorescence signals and to minimize the instrument’s volume, both separated optical components and optical fibers were used [24]. The weight of the instrument was 22 kg in water, so it was convenient to mount on the bottom sink platform for in situ detection. For the stability and reliability of the instrument, a 266 nm, LD-pumped, all-solid-state UV laser (MPL-W-266, pulse width ≤5 ns, beam diameter at the aperture ≤2 mm, full-angle beam divergence <2 mrad, output wavelength 266 ± 1 nm, and pulse energy 5–30 μJ) was used as the excitation module of the LIF device, because of its short wavelength, high resolution, and concentrated energy [36]. Compared with traditional lasers, such as gas lasers, ion lasers, helium–cadmium lasers [36], and other solid-state lasers (such as 445 nm, 532 nm, and 635 nm), we found that the 266 nm, LD-pumped, all-solid-state UV laser was more suitable for the detection of aromatic amino acids. The excited beam passed through the specially designed waterproof, pressure-resistant, and transparent glass window and excited the seawater in the sample pool (Figure 2). The fluorescence emitted from the sample was concentrated with a mirror and convex lens (focal length of 75 mm), and was detected with a scientific, high-sensitivity spectrometer (Ocean Optics QE pro 6500; spectrum range 200 nm to 1100 nm and spectral resolution 0.8 nm). The shell of the instrument, made of aluminum 7075, withstood 30 MPa of pressure in the deep sea. Two optical windows were mounted on the shell of the instrument so as to excite and receive light signals. The control module governed the operation of the instrument and the preprocessing and storage of the data. When the LIF instrument was working normally, it sent a signal to the operating platform every minute. If a problem occurred, the signal was interrupted, allowing the operator to quickly confirm the operation of the instrument status. In shallow water, the instrument was used for in situ on-line detection (0–5 m, shown in Figure 3); in deep water (5–1000 m), the self-contained sampling mode opened and the stored data was transmitted to an external storage device for further processing once the instrument was raised above the surface.

### 2.2. Quantifying Aromatic Amino Acids with Fluorescence

As the aromatic amino acids were excited by the UV laser (266 nm), the positions of their fluorescence peaks varied for differentiation [37]. After being excited, the fluorescence intensity was proportional to the concentration of the aromatic amino acid under the given conditions, and was therefore used to quantify the concentrations [38].

## 3. Results

### 3.1. Different Aromatic Amino Acids Can Be Distinguished

**Figure 4:**
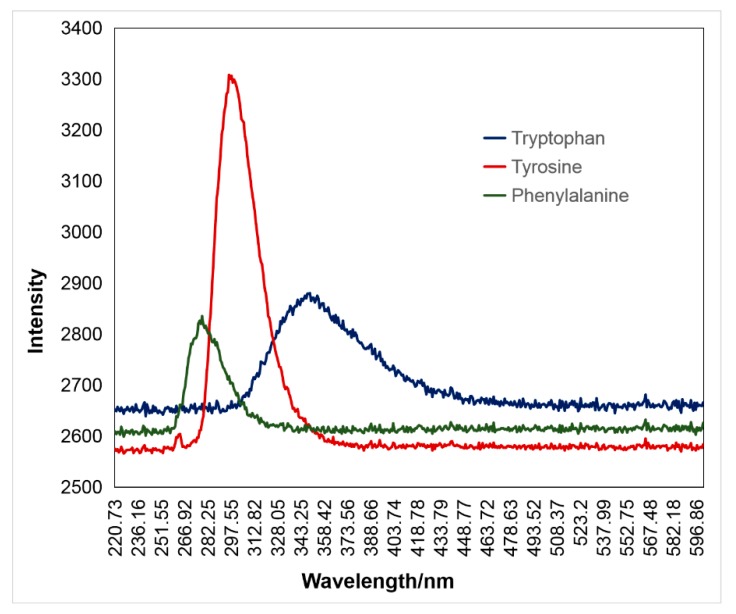
Fluorescence of three aromatic amino acids excited by a UV laser at 266 nm. Tryptophan fluoresced to a maximum of 350 nm, tyrosine to a maximum of 300 nm, and phenylalanine to a maximum of 280 nm.

LIF is a widely used technique in protein analysis, which can detect intrinsic fluorescence because the presence of aromatic amino acids [39,40]. It is an indirect absorption technique because photons emitted spontaneously are recorded as signals when the species absorbs the incident laser light [41]. Therefore, information about the sample can be obtained from the wavelength-dispersed fluorescence. When excited by the 266-nm UV laser, the fluorescence peaks of aromatic amino acids tryptophan, tyrosine, and phenylalanine were located at 350 nm, 300 nm, and 280 nm, respectively (Figure 4 and Table 1). The results showed that this method was favorable for distinguishing the detected aromatic amino acids.

### 3.2. Linear Relationship between Amino Acid Concentrations and Fluorescence Intensity

**Figure 5:**
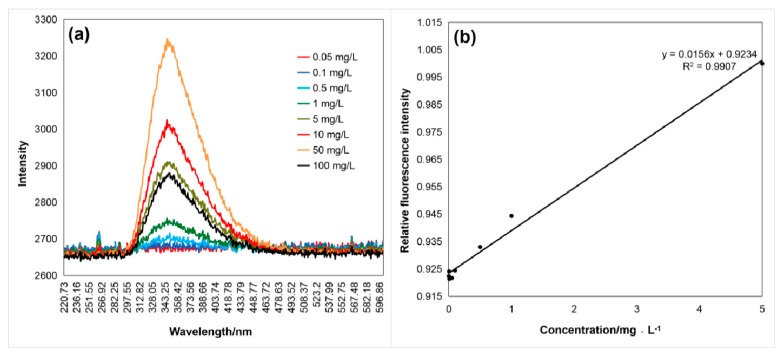
(**a**) Fluorescence spectrum (λ_max1_ = 350 nm) of tryptophan excited by the 266 nm laser, (**b**) relation curve between the normalized fluorescence peak intensity, *I_F_*_1_, and the tryptophan concentration, *C*_1_, of 0.05–100 mg/L with a coefficient of determination of 0.9907.

**Figure 6:**
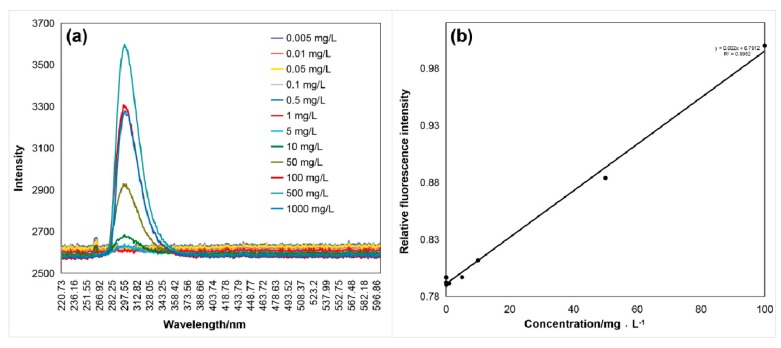
(**a**) Fluorescence spectrum (λ_max2_ = 300 nm) of tyrosine excited by the 266 nm laser, and (**b**) relation curve between the normalized fluorescence peak intensity, *I_F_*_2_, and the tyrosine concentration, *C*_2_, of 5 ug/L–1 g/L with a coefficient of determination of 0.9952.

**Figure 7:**
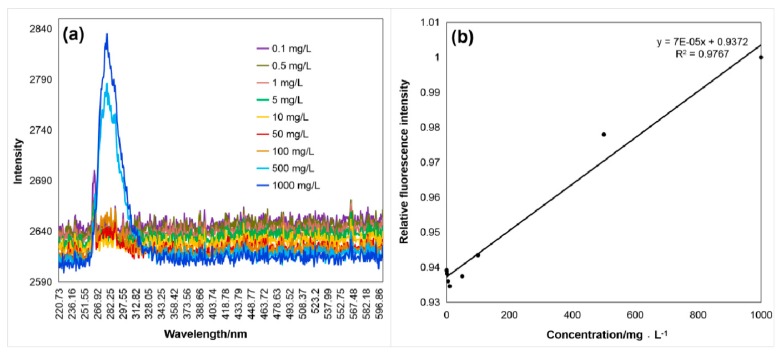
(**a**) Fluorescence spectrum (λ_max3_ = 280 nm) of phenylalanine excited by the 266 nm laser, and (**b**) the relation curve between the normalized fluorescence peak intensity, *I_F_*_3_, and the phenylalanine concentration, *C*_3_, of 0.1 mg/L^−1^ g/L with a coefficient of determination of 0.9767.

As shown in Figure 5, Figure 6 and Figure 7, the fluorescence intensity linearly increased with the aromatic amino acid concentration with the excitation by the 266 nm UV laser. Figure 5a, Figure 6a and Figure 7a show the raw data of spectra with the fluorescence peaks of different concentrations of the aromatic amino acids. The relationship between fluorescence intensity and concentration, after simple data processing, is shown in Figure 5b, Figure 6b and Figure 7b. The fluorescence peak data of the three aromatic amino acids were normalized to facilitate comparative analysis. The linear relationship between the concentration and normalized fluorescence intensity was retrieved, as follows:

(1) $$I_{F_{1}}=0.01560c_{1}+0.9234$$

(2) $$I_{F_{2}}=0.00200c_{2}+0.7912$$

(3) $$I_{F_{3}}=0.00007c_{3}+0.9372$$

In Equations (1)–(3), *I_F_* is the amino acid normalized fluorescence intensity and *C* is the sample concentration (mg·L^−1^); *C*_1_, *C*_2_, and *C*_3_ represent the tryptophan, tyrosine, and phenylalanine concentration, respectively.

These regression results show the high performance for the detection, identification (Table 1), and quantitative analysis (shown in Equations (1)–(3)) of the aromatic amino acids. The concentration was reversed from the fluorescence intensity of the amino acid and the normalization formula (coefficient of determination reached more than 97%), and the process was relatively simple.

## 4. Discussions

### 4.1. The Equipment Can Execute Different Types of In Situ Observations

The equipment designed in the present study can execute different types of in situ observations, namely: (1) fixed station observations, where the instrument can be fixed at one station for long-time observation, and (2) towed by ships, where the equipment can be designed for a vehicle to be towed by a ship for moving observations. The spatial and temporal distribution of aromatic amino acids can vary, and the equipment designed for executing different types of in situ observations can satisfy different requirements.

### 4.2. Detection Limit of the Instrument Can Satisfy Most Natural Water

The LIF instrument can be used in most natural water. The lowest detectable concentrations of tryptophan, tyrosine, and phenylalanine were 4.70 × 10^−9^ mol·L^−1^, 2.76 × 10^−8^ mol·L^−1^, and 6.05 × 10^−7^ mol·L^−1^, respectively. It is believed that concentrations of amino acids in seawater from 10^−6^ to 10^−7^ mol·L^−1^ can be measured using the LIF instrument. Additionally, we compared the amino acid concentrations with the following results: (1) Chau and Riley [44] analyzed the results of previous studies, and concluded that the concentration of 11 amino acids in seawater ranged from 2 to 6 μg·L^−1^. (2) The average concentration of THAAs in the East China Sea was (9.5 ± 4.2) × 10^−7^ mol·L^−1^ (in the range of 4.46 × 10^−7^ mol·L^−1^ to 2.25 × 10^−6^ mol·L^−1^) [45]. (3) The average DAA values in North Taihu Lake, South Taihu Lake, and East Taihu Lake were (2.59 ± 0.71) × 10^−6^ mol·L^−1^, (4.8 ± 1.4) × 10^−7^ mol·L^−1^, and (4.8 ± 1.6) × 10^−7^ mol·L^−1^, respectively [17]. A comparison of the results confirmed the advantages of this detection instrument when quantifying in situ the concentrations of the three amino acids for varied water environments.

### 4.3. The Equipment Is More Suitable for Working in the Deep Sea

To our knowledge, few in situ observation methods have been developed to detect aromatic amino acids in deep seas. The limits of the conventional methods are that the samples can only be analyzed in the laboratory, and that the content and properties of the biological macromolecules in the water might be affected by the sampling, transportation, and pretreatment. These pretreatments can change external conditions, such as the temperature and pressure of the samples, which is time-consuming and cumbersome, and the amino acid content in the water may fluctuate. In the deep sea, with the high pressure and large difficulties in sampling, in situ observations can run uninterruptedly for a long time. In addition, the dark environment of the deep sea can provide excellent signals without light pollution.

### 4.4. Fluorescence Quenching of Tryptophan and Tyrosine at High Concentrations

**Figure 8:**
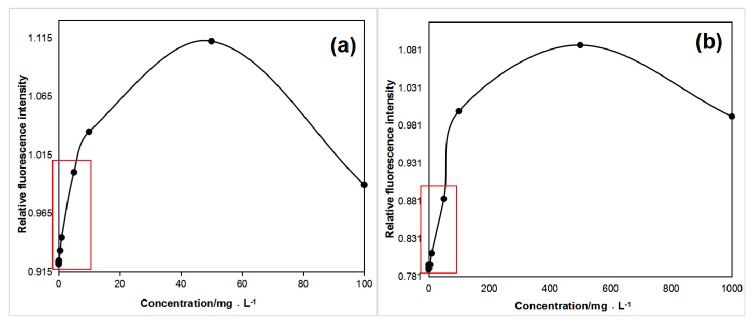
Fluorescence quenching of (**a**) tryptophan and (**b**) tyrosine.

With the increased concentration of the aromatic amino acid solution, the fluorescence quantum yield of tryptophan and tyrosine first increased and then decreased (Figure 8) [46], and notable fluorescence quenching was observed [41,47]. The part marked with a red square in Figure 8a,b is shown in Figure 5b and Figure 6b. If the tryptophan concentration was greater than 10 mg/L, the above concentration fluorescence quenching effects occurred. However, tyrosine quenching effects were found above 150 mg/L, and no fluorescence quenching of phenylalanine was observed. Because fluorescence quenching rarely occurs in natural water, we did not discuss it in detail in the paper.

## 5. Conclusions

LIF instruments are portable, sensitive, and reproducible for the detection and quantification of aromatic amino acids. The instrument weighs only 22 kg in water, making it easy to carry in the field, and its data processing method and process are simple and fast. Moreover, its minimum detectable concentrations of tryptophan, tyrosine, and phenylalanine are 4.70 × 10^−9^ mol/L, 2.76 × 10^−8^ mol/L, and 6.05 × 10^−7^ mol/L, respectively. In addition, the concentration of the amino acid may be further calculated from its fluorescence intensity. Most importantly, LIF enables the in situ detection of aqueous amino acids; this is a unique advantage of this technology compared with the current water environment amino acid detection methods. It also has a very broad detection range for rivers, lakes, reservoirs, etc. Additionally, the pressure-resistant design of the system ensures withstanding in situ detection at 3000 m below the sea surface. The method reported here can improve the efficiency of detecting aromatic amino acids in water environments and provides a practical detection method.

## Figures and Tables

**Table 1:** Emission maxima of fluorescence spectra of aromatic amino acids [21,37,42,43].

Analyte	Laser Excitation Wavelength (nm, ±1 nm)	Maximum Fluorescence Emission Wavelength (λ_max_, nm, ±5 nm)	Reference
Tryptophan	280	350	[21,22,37,42,43]
	266	350	
Tyrosine	275	300	[21,37,42]
	266	300	
Phenylalanine	260	280	[42]
	266	280	[40]
	255	280	[21]

## Abbreviations

1	**UV**	Ultraviolet
2	**LIF**	Laser-induced fluorescence
3	**THAAs**	Total hydrolyzed amino acids
4	**DON**	Dissolved organic nitrogen
5	**POM**	Particulate organic matter
6	**DOM**	Dissolved organic matter
7	**SOM**	Sedimentary organic matter
8	**DFAAs**	Dissolved free amino acids
9	**D/L**	Ratio of D-enantiomers to L-enantiomers
10	**HPLC**	High performance liquid chromatography
11	**GC**	Gas chromatography
12	**DAA**	Dissolved amino acids
13	**IEC**	Ion exchange chromatography
14	**VIS**	Visible spectrophotometer
15	**MS**	Mass spectrometer
16	**CE**	Capillary electrophoresis
17	**LC**	Liquid chromatography
18	**ELSD**	Evaporative light scattering detector
19	**IPAD**	Integrated pulsed amperometric detection
20	**LD-pumped**	Laser diode pumped

## Appendix B

**Table A1:** Comparison of amino acid detection methods.

Classification	Name	Advantages	Disadvantages	In Situ Detection
**Indirect analysis (derivative method)**	**IEC**	Post-column ninhydrin-derived IEC can simultaneously detect primary and secondary amino acids, and is suitable for the analysis and detection of amino acids in complex samples.	The conditions for the derivatization reaction are demanding, and a heating derivative device is required; the analysis process is long, the detection cost is high, and it will cause serious glycine contamination.	No in situ detection
	**UV–VIS**	In visible light detection, the sensitivity of the ninhydrin-derived and DABSYL-Cl color reaction is high, the stability of the derivative is good, and the latter is simpler and more efficient [49]. In UV detection, the OPA derivatization agent itself does not interfere with separation and detection, the derivatization operation is simple, and the sensitivity and repeatability are good [50].	Although there are many types of derivatizing agents, none of the derivatizing agents are fully suitable for the analysis of all amino acids [51]. It is necessary to select different derivatizing agents for the types of amino acids to be tested; only the total amount of one or a class of amino acids can be determined, and the separation and analysis of amino acids cannot be performed [52].	
	**GC**	High performance, good selectivity, high sensitivity, and simple operation.	Derivative conditions are harsh: unstable and non-volatile materials cannot be separated, and because of the characteristics of different amino acids (such as the rate of derivatization or the different derivatization reagents), the determination of all of the amino acids cannot be performed using the same column. In addition, the sample needs to be desalted, and the operation of the experiment is cumbersome and can cause pollution [53].	
	**GC–MS**	More efficient than GC.		
	**RP-HPLC**	Pre-column derivation overcomes the disadvantages of post-column derivation operations, and has a high sensitivity, fast analysis, and diverse reagents.	Different derivatizing reagents have their own shortcomings [32], and often problems such as unstable derivatives, cumbersome reaction conditions, and long operating times are not conducive to the rapid analysis of amino acids [30]. The derivative process cannot be replaced by an instrument, it must be done manually, and the cost of detection is high [54].	
	**CE**	High separation efficiency, faster analysis, no gradient elution, wide range of applications, small sample size, simple instrument, and low cost [55].	Still need to use chemical derivation technology, is time-consuming and laborious, and has a reduced accuracy.	
	**LC–MS**	High sensitivity and selectivity, able to provide sufficient sample structure information, no complicated pre-processing or derivatization of the sample, high detection efficiency, and good anti-interference performance.	Instruments are expensive and costly to test.	
**Direct analysis**	**LC–ELSD**	The response of ELSD does not depend on the optical properties of the sample. It can directly detect the properties of the material without UV absorption or fluorescence functional groups, and is not affected by its functional groups. Any sample with a lower volatility than the mobile phase can be detected [52], no derivative is required, there are a wide range of applications, and it is suitable for the rapid determination of amino acids.	Low detection sensitivity, high detection limit, and cannot solve the problem of detection of trace amino acids well.	
	**IPAD**	No cumbersome sample preparation steps, you can directly measure the sample by diluting it to the appropriate concentration, and it has a high accuracy and sensitivity.	High requirements for experimental operations (instruments and experimenters) [56].	
	**LIF**	Each substance has a corresponding “fingerprint” fluorescence spectrum. A wide range of detectability, a low detection limit, no complicated derivative process, no damage to the sample, it is portable and efficient, has an ultra-high sensitivity [57], and has in situ monitoring.	In situ detection technology is still immature, it has fluorescence absorption and quenching, the detection accuracy is limited by the instrument signal-to-noise ratio, etc.	In situ detection
